# A case of Poland Syndrome associated with dextroposition

**DOI:** 10.1186/1824-7288-36-21

**Published:** 2010-02-20

**Authors:** Doriana Lacorte, Maria Marsella, Pietro Guerrini

**Affiliations:** 1 Neonatal Intensive Care Unit, Department of Clinical and Experimental Medicine, Pediatrics, University of Ferrara, Italy

## Abstract

Classical Poland Syndrome (PS) is characterized by unilateral, partial or complete absence of the sternocostal head of the major pectoral muscle and brachysyndactyly of fingers on the same side.

We report the case of a newborn infant with dextrocardia and PS located on the left side.

This association is very rare: to date only 19 cases have been described in scientific literature. In all reported cases, as in the present, the Poland defect involved the left side and was associated to rib defects, whereas most cases of PS are on the right side and few have rib defects. This case supports the view that dextrocardia follows the loss of volume of the left hemithorax caused by Poland sequence and that the combination of PS and dextrocardia is not coincidental.

## Introduction

PS is a rare congenital anomaly classically consisting of the combination of unilateral aplasia/hypoplasia of the sternocostal head of the major pectoral muscle and ipsilateral brachysyndactyly [1-3]. Other usual anomalies in PS are malformations of the anterior chest wall and breast. Dextrocardia, lung herniation, renal, vertebral and lower limb malformations have been described in rare cases [4]. Moreover, reports of PS associated with other known syndromes in the same individual are not uncommon. In fact, associations with Moebius Syndrome, facio-auricolo-vertebral dysplasia and frontonasal dysplasia have been described [5].

The incidence is 1:30.000, with a higher frequency among males [4]. In 75% of the unilateral cases it is located in the right hemithorax [2]. Reports of bilateral agenesis of the muscle have been infrequently reported in literature [4,5]. Most cases of PS reported are sporadic; however familiar cases have been occasionally described. The possible mechanism suggested is a paradominant inheritance in which mutations can be transmitted through many generations in the absence of an apparent phenotype [5].

The cause of PS is unknown; however, it is believed that in the embryonic development, during the sixth week of pregnancy, a momentary interruption or reduction in the circulation of the subclavian and vertebral arteries of one of their peripherical ramifications primes the pathogenetic mechanism of the syndrome and results in different degrees of severity depending on the length and intensity of the vascular interruption [2].

The diagnosis is generally postnatal. There are only two reports in which the diagnosis is made prenatally. In case of a dextroposition in the fetus, it would be important to consider PS in the differential diagnosis. In these cases, sonographic evaluation should also include a focused examination of the rib cage [6].

Because clinical features are highly variable and not all present in the same individual, patients with PS should undergo an accurate physical examination and investigations to exclude renal, cardiac, or other important anomalies.

Patients with significant deformities of the chest wall and overlying soft tissue may need surgical reconstruction, generally recommended after the completion of growth. Emergency surgery is reserved for a very rare subset of children with compromised respiratory function [4].

## Case report

We report the case of a neonate born by caesarean section at 35 weeks of gestation because of maternal dilatative myocarditis. Birth weight was 2.5 Kg (25-50th percentile), length was 49 cm (75 - 90th percentile), occipitofrontal circumference was 33 cm (50 - 75th percentile), and Apgar score were 7 and 8 at the 1st and 5th minute. At birth he presented respiratory depression, was treated with nasal continuous positive airway pressure, and admitted to our NICU. Respiratory depression resolved in two hours and subsequently the patient did not present other considerable clinical problems.

**Figure 1:**
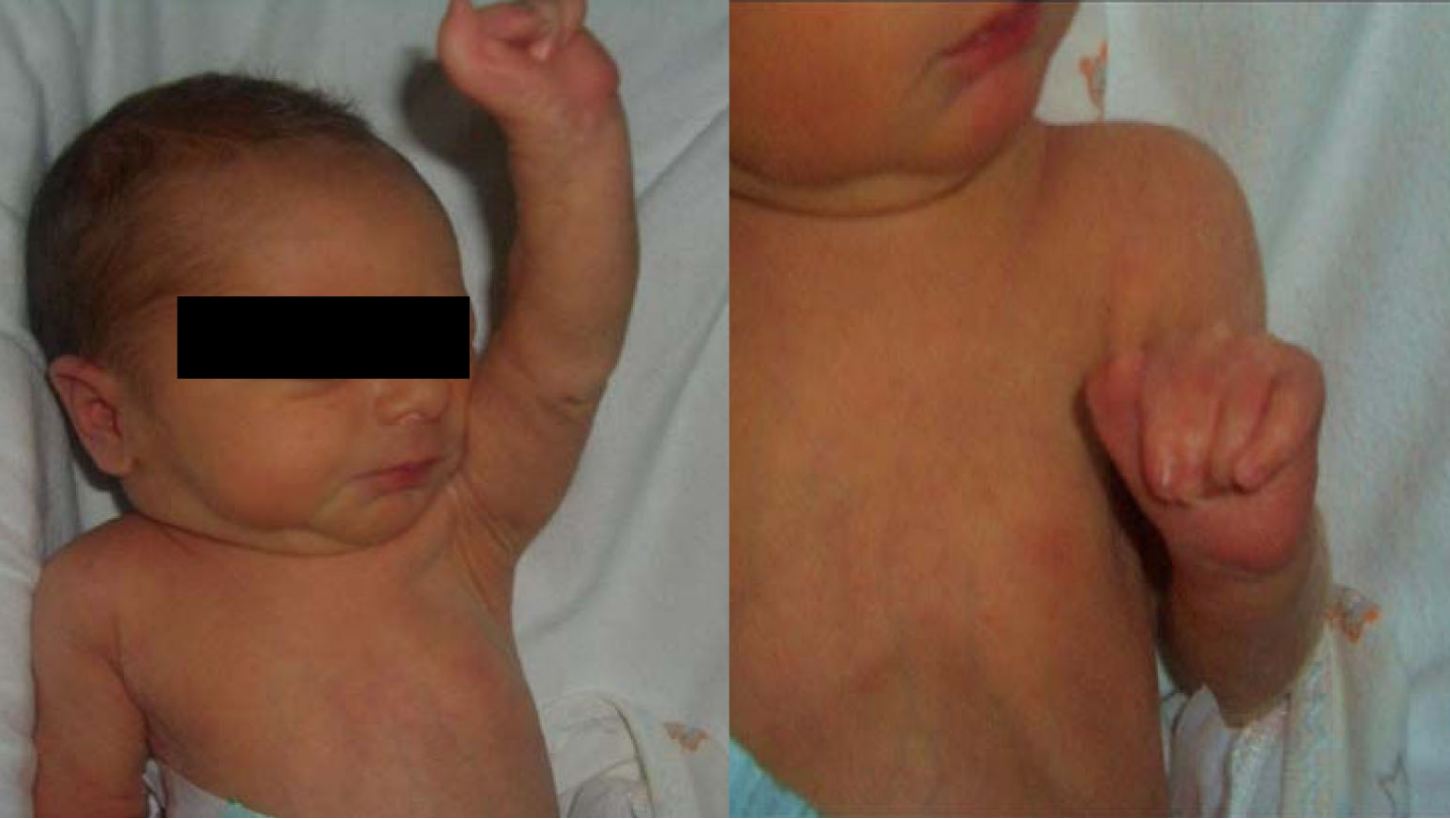
**Depression of the left anterior chest wall (1a); brachysyndactyly of the second, third and fourth finger of the left hand (1b)**.

Initial physical examination showed depression of the left anterior chest wall (figure 1a), centralized cardiac apex and brachysyndactyly of the second, third and fourth finger of the left hand (figure 1b).

**Figure 2:**
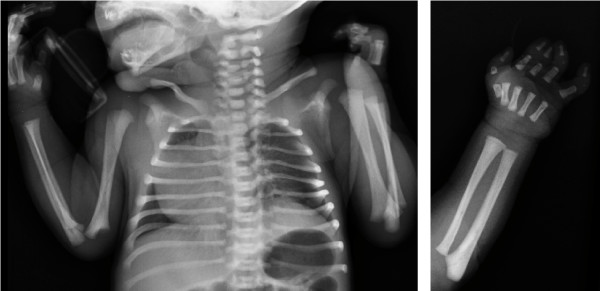
**Chest X-ray: asymmetric chest with reduction of the third to fifth left intercostal spaces and dextrocardia (2a)**. **Left hand X-ray**: hypoplasia of the main phalanx of the I finger, absence of the intermediate phalanx of the II and III fingers, and substitution of the intermediate phalanx of the IV and V fingers with a small ossification nucleous (2b).

Radiological examination revealed an asymmetric chest with reduction of the third to fifth left intercostal spaces and dextrocardia (figure 2a). Rx of the left hand showed hypoplasia of the main phalanx of the thumb, absence of the intermediate phalanx of the forefinger and middle finger, and substitution of the intermediate phalanx of the annular and little finger with a small ossification nucleous (figure 2b). Ultrasound of the chest showed hypoplasia of the left pectoralis and confirmed dextrocardia without evidence of cardiac or great vessel malformations.

These anomalies were compatible with the diagnosis of PS.

Further systemic evaluation, including examination of lower limbs, hair and nails, did not show other anomalies. He demonstrated a normal range of joint movements, except for the affected hand. Neurological examination was normal. Renal ultrasound excluded important anomalies which can occur in PS. Karyotype was 46, XY.

Autoimmune hypothyroidism, antiphospholipid syndrome and thrombophilia in the mother, lupus erythematosus in the grandmother were present in the familiar medical history.

## Discussion

To date the association of dextrocardia and PS has been described only in 19 cases [3]. In all these cases, as in ours, the syndrome was left sided and associated with rib defects, which occur only in about 15% of patients with pectoral defects on the right [7,8]. Dextrocardia associated with PS is always an isolated dextroposition: the normally connected heart is simply displaced to the right [3,7,8].

Because both isolated dextrocardia and PS are very rare, in accordance with other Authors [6-9], we believe that the relationship between dextrocardia and PS is not a coincidence; in particular dextrocardia might follow the loss of volume of the left hemithorax caused by the development of the Poland sequence. In a recent study [10], all patients with left sided PS and partial agenesis of two or more ribs presented dextrocardia, whereas it was never associated with partial agenesis of a single rib. These findings suggest that mechanical factors during embryonic life could explain the association between left-sided PS and dextrocardia; in particular partial agenesis of 2 or more ribs is needed to displace the heart towards the right side. The fact that dextrocardia in PS is neither associated with situs inversus, nor with other complex anomalies, further supports this hypothesis.

We have no data to correlate the autoimmune diseases in the relatives with the PS in the proband. Because the presence of antiphospholipid antibodies and thrombophilia in the mother could increase the risk of thrombotic complications and obstructive vascular disease in the fetus [11,12], it could be hypothesized that the autoimmune disorders described in the relatives could explain PS in the fetus. However, scientific literature does not confirm this association and the hypothesis needs to be confirmed in other patients.

## Consent

Written informed consent was obtained from the parents of the patient for publication of this case report and accompanying images. A copy of the written consent is available for review by the Editor-in-Chief of this journal.

## Competing interests

The authors declare that they have no competing interests.

## Authors' contributions

PG defined the clinical picture of the patient and formulated the diagnostic suspicion of the Poland Syndrome. DL and MM were involved in the collection of clinical data of the patient and in drafting the manuscript. All authors read and approved the final manuscript.
